# Evaluating gene × gene and gene × smoking interaction in rheumatoid arthritis using candidate genes in GAW15

**DOI:** 10.1186/1753-6561-1-s1-s17

**Published:** 2007-12-18

**Authors:** Ling Mei, Xiaohui Li, Kai Yang, Jinrui Cui, Belle Fang, Xiuqing Guo, Jerome I Rotter

**Affiliations:** 1 Department of Medical Genetics, Cedars-Sinai Medical Center, 8700 Beverly Boulevard, 665 West Tower, Los Angeles, California 90048, USA

## Abstract

We examined the potential gene × gene interactions and gene × smoking interactions in rheumatoid arthritis (RA) using the candidate gene data sets provided by Genetic Analysis Workshop 15 Problem 2. The multifactor dimensionality reduction (MDR) method was used to test gene × gene interactions among candidate genes. The case-only sample was used to test gene × smoking interactions. The best predictive model was the single-locus model with single-nucleotide polymorphism (SNP) rs2476601 in gene *PTPN22*. However, no clear gene × gene interaction was identified. Substantial departure from multiplicativity was observed between smoking and SNPs in genes *CTLA4*, *PADI4*, *MIF*, and SNPs on chromosome 5 and one haplotype of *PTPN22*. The strongest evidence of association was identified between the *PTPN22 *gene and RA status, which was consistently detected in single SNP association, gene × gene interaction and gene × smoking interaction analyses.

## Background

Rheumatoid arthritis (RA) is a complex autoimmune disease. The etiology of the disease is not clearly understood. Risk factors of RA include genetic factors, race (Native American), female gender, obesity, old age, and smoking [1,2]. However, like most complex diseases, few studies of gene × gene interaction and gene × environmental interaction have been performed because a large sample size is required to identify such effects in traditional statistical paradigms. Logistic regression is commonly used in detecting interactive effects between genes or environmental factors in epidemiologic studies. However, the parameters cannot be accurately estimated when there are many independent variables while the sample size is not large enough [3]. Recently, Ritchie et al. [4] introduced a multifactor dimensionality reduction (MDR) method for identifying gene × gene interaction or gene × environmental interaction to overcome this limitation of traditional logistic regression [3-5]. This approach enumerates all possible combinations of genotype or environmental factors associated with high risk and low risk of disease, and it may enable us to find interactions between genes in the absence of main effects [3-5].

To detect potential epistasis in RA, we evaluated 1) disease associations using single SNPs (single-nucleotide polymorphisms) from 15 candidate genes and haplotypes of the *PTPN22 *gene, 2) gene × gene interactions among the candidate genes using the MDR method and logistic regression, and 3) gene × environmental (smoking) interactions using a case-only study design.

## Methods

### Materials

The data sets for the candidate gene studies of RA were provided by Genetic Analysis Workshop 15 (GAW15) Problem 2. There were two case-control data sets. The first one included 855 unrelated controls and 839 cases, as well as genotype data on 20 SNPs from 15 candidate genes, which were selected from previously published associations with RA or other autoimmune disorders by Plenge et al. [6]. The second data set included 1519 unrelated controls and 1393 cases, and genotype data on 14 SNPs from the *PTPN22 *gene. Additional phenotype data, including smoking history, age of onset, sex, and body mass index, were available for cases only in both data sets. There were 408 and 720 affected sibling pairs among cases in the two data sets, respectively.

### Statistical analysis

Single SNP and haplotype (*PTPN22 *only) associations with disease status were first evaluated. To account for the dependency among family members, the generalized estimating equations methods (GEE1) [7] as implemented in the GENMOD procedure of SAS 9.0 was utilized in the association analysis by using family as the cluster factor, i.e., members from the same family were assumed to be correlated and those from different families were assumed to be independent. The haplotype block structure of *PTPN22 *was evaluated by Haploview [8]. Individual haplotypes were reconstructed using the PHASE 2.0 by assigning each haplotype with maximum probability [9]. Seventy-four percent of haplotype assignments had probabilities of 100% and 93% had probabilities of 80% or better. Individuals whose haplotype assignment had probability below 80% were excluded from subsequent analysis. Association analysis was carried out for each common haplotype in turn. For each haplotype, a dominant model was assumed, i.e., carriers of the particular haplotype versus non-carriers were compared for their RA status.

To test gene × gene interactions, MDR was used to determine the genetic model that could most successfully predict the disease status or phenotype from several loci. SNP rs2240340 on the *PADI4 *gene was excluded from analysis due to its large amount of missing data. One thousand three hundred and thirty case-control samples with completed marker data on 19 SNPs from 14 candidate genes were utilized in the MDR analysis. Cross-validation (CV) consistency and balanced accuracy estimates were calculated for each combination of a pool of genetic polymorphisms. The model with the highest accuracy and maximal CV was considered to be the best [5]. We determined statistical significance by comparing the accuracy of the observed data with the distribution of accuracy under the null hypothesis of no associations derived empirically from 1000 replicates of permutations [10]. The null hypothesis was rejected when the *p*-value derived from the permutation test was 0.05 or less. As a follow-up, logistic regression analysis was conducted if there was suggestive interaction.

We also examined the interaction between SNPs and smoking history in RA cases. The logistic function in the GENMOD procedure was used to quantify departure from multiplicativity. Odds ratios and 95% CIs were estimated. To adjust for multiple tests, empirical *p*-values were obtained from 1000 permutations. For the *PTPN22 *gene, interaction effects between *PTPN22 *haplotypes and smoking among cases were evaluated for RA status.

## Results

### 1. Single SNP and *PTPN22 *haplotype association

Table 1 lists the association analysis results between disease and individual markers. One SNP from each of the five genes, *HAVCRI*, *CTLA4*, *SUMO4*, *MAP3K7IP2*, and *PTPN22*, were found significantly associated with RA.

**Table 1:** Association between SNPs and RA

Candidate gene	SNP	*p*-value (11 vs. 12 vs. 22)	*p*-value (11/12 vs. 22)
*HAVCRI*	5509_5511delCAA	0.066	**0.034^a^**
*HAVCRI*	rs6149307	0.189	0.068
*CTLA4*	CT60	**0.016**	**0.005**
*CARD15*	HugotSNP12ms3	--^b^	0.754
*CARD15*	HugotSNP8ms2	0.838	0.553
*CARD15*	Hugot_SNP13ms2	--	0.695
Chr 5	IGR2096ms1	0.473	0.243
Chr 5	IGR3084ms1	0.819	0.713
Chr 5	IGR3138ms1	0.861	0.732
*IL3*	rs31480	0.618	0.384
*SUMO4*	rs237025	**0.0003**	**<0.0001**
*MAP3K7IP2*	rs577001	**0.002**	**0.001**
*MIF*	rs755622	0.842	0.979
*TNFRFF1b*	rs1061622	0.704	0.684
*DLG5*	rs1248696	0.269	0.129
*SLC22A4*	rs2073838	0.904	0.771
*PADI4*	rs2240340	0.574	0.330
*IL4*	rs2243250	0.311	0.147
*RUNX1*	rs2268277	0.55	0.583
*PTPN22*	rs2476601	**<0.0001**	**<0.0001**

Allele "1" is the putative susceptibility allele, and allele "2" is the non-susceptibility allele

^a^Bold text indicates *p *< 0.05.

^b^--, Results are not available because there is no homozygote "11" in some groups.

Five common haplotypes of the *PTPN22 *with frequency >10% were constructed. Of the two haplotypes with significant associations with RA, one was a risk haplotype (11222221122221; 1: minor allele, 2: major allele; frequency: 11.6%), with a higher carrier frequency in cases than in controls (30.0% vs. 14.9%, *p *< 0.0001); whereas the other was protective (22122222222222; frequency: 10.9%), with a lower carrier frequency in cases than in controls (16.4% vs. 24.7%, *p *< 0.0001).

### 2. Gene × gene interaction

Table 2 lists the results from MDR. The one-locus model with SNP rs2476601 on gene *PTPN22 *had a maximum test accuracy (*p *= 0.004) and a maximum CV consistency of 10 out of 10, indicating that this was the best model. The second-best model was a two-locus model consisting of rs1248696 on the *DLG5 *gene and rs2476601 on *PTPN22 *(*p *= 0.013). The combination of rs1248696_22 and rs2476601_22 was associated with being in the low-risk group when compared to others (OR = 0.46, 95%CI: 0.36, 0.60). However, we could not confirm the interactive effect between these two markers in the follow-up logistic regression analysis under the GEE model. No better models were identified for three and/or more locus models.

**Table 2:** Multilocus interaction model for RA selected from MDR

Model	Balanced accuracy	CV consistency	*p*-value
rs2476601 (*PTPN22*)	0.5747	10/10	**0.004^a^**
rs2476601 (*PTPN22*) rs1248696 (*DLG5*)	0.5705	8/10	**0.013**
rs2476601 (*PTPN22*) rs6149307 (*HAVCRI*) rs2243250 (*IL4*)	0.5534	7/10	0.09
rs2476601 (*PTPN22*) IGR2096ms1 (chr 5) rs237025 (*SUMO4*) rs2268277 (*RUNX1*)	0.5243	6/10	0.475

^a^Bold text indicates *p *< 0.05.

### 3. Gene × smoking interaction

Two categories of environmental exposure, ever smoked and current smoking, were used to test for gene × environmental interactions. No significant departure from multiplicativity was observed between current smoking and markers. Interactive effects with ever smoking were found in the primary analysis for five SNPs, including CT60 on the *CTLA4 *gene, rs2240340 on *PADI4*, IGR3084ms1 and IGR3138ms1 on chromosome 5, and rs755622 on the *MIF *gene. The empirical *p*-values derived from the 1000 permutations were similar to the nominal ones (Table 3).

**Table 3:** Gene × smoking interactions

		Ever smoked					
						
Marker	Haplotype	No	Yes	OR_int_	95%CI	*p*-Value	Empirical *p*
CT60 (*CTLA4*)	11	67	58				
	12/22	289	393	1.69	1.11, 2.55	0.025	0.023
rs2240340 (*PADI4*)	11	40	29				
	12/22	122	162	2.76	1.24, 6.16	0.026	0.019
IGR3084ms1 (chr 5)	11	23	47				
	12/22	327	393	0.59	0.36, 0.97	0.039	0.042
IGR3138ms1 (chr 5)	11	36	69				
	12/22	314	374	0.62	0.40, 0.96	0.033	0.04
rs755622 (*MIF*)	22	234	329				
	11/12	120	128	0.73	0.53, 0.99	0.046	0.052

One of the common haplotype of *PTPN22 *(22222222211221, frequency: 18%) was found to interact with ever smoking at borderline significant level (OR = 0.78, 95%CI: 0.60–1.01, *p *= 0.06); however, the risk and the protective haplotypes that were identified previously in the case-control sample did not show any departure from multiplicativity with smoking in the case-only study.

## Discussion

We explored gene × gene and gene × smoking interactions using the candidate gene data set provided by GAW15. The best predictive model for RA status is the single-locus model containing rs2476601 on gene *PTPN22*. SNP rs2476601 is a well known functional SNP that is associated with increased risk of RA. The best combination model selected by MDR consisted of rs2476601 on *PTPN22 *and rs1248696 on *DLG5*. However, the susceptibility interaction was not confirmed in the following logistic regression analysis. The possible reason for the inconsistent results is that in MDR, we actually did not test statistical interaction which was defined as 'deviation from multiplicativity' as in logistic regression. The significant results from MDR only implies that the combination of the markers contributes to an increased or decreased risk of disease and the effect between the markers could be either multiplicative or deviation from multiplicative.

The case-only study has its particular advantage in testing gene × environmental interaction and it requires smaller sample size [11]. It allows us to test interactive effects in the absence of the information from controls under the assumption that the two risk factors are independently distributed in the population at risk [10]. In GAW15 Problem 2, we used this design to identify a gene × smoking interaction in RA because no smoking information was available from controls. We assumed genetic polymorphism and smoking exposure are independent of one another in controls. Substantial departure from multiplicativity was observed between ever smoking and markers from *CTLA4*, *PADI4*, *MIF*, and chromosome 5. Among these markers, only SNP CT60 from gene *CTLA4 *showed a main effect with RA in the single SNP analysis. One possible explanation for this phenomenon is that the existence of gene × smoking interactions could mask the true genetic effect if we only test the marginal association, especially when the gene status modifies the smoking effect in the opposite directions in the total sample. Another possible explanation is the difference in the tested samples: only cases were used in the gene × smoking interaction studies, while the single SNP association was evaluated in the case-control sample.

*PTPN22 *has been reported to be associated with RA [6,12]. In this study, we tested single gene association, gene × gene interactions and gene × smoking interactions using three different methods. In single SNP analysis, *PTPN22 *showed the strongest association with RA status (*p *< 0.0001). In the following gene × gene interaction analyses by MDR, both the best single and the best combined models included *PTPN22 *gene. Furthermore, haplotype analysis using the second data set identified two haplotypes of the *PTPN22 *associated with RA and more importantly, there was a trend toward interaction between this gene and smoking. Therefore, the consistent findings here provide further evidence of the genetic involvement of *PTPN22 *in the etiology of RA.

## Conclusion

In conclusion, our analyses confirmed the role of genetic and environmental factors in rheumatoid arthritis. Strong evidence of association was identified for the *PTPN22 *gene, which was observed in all three analyses. Other genes (*HAVCRI*, *CTLA4*, *SUMO4*, *MAP3K7IP2*, *PAID4*, chromosome 5 locus, *MIF*) may also contribute to the development of rheumatoid arthritis directly or within the context of smoking.

## Competing interests

The author(s) declare that they have no competing interests.
